# High-Spin Manganese(V) in an Active Center Analogue of the Oxygen-Evolving Complex

**DOI:** 10.1021/jacs.4c14543

**Published:** 2025-02-19

**Authors:** Olesya S. Ablyasova, Mihkel Ugandi, Esma B. Boydas, Mayara da Silva Santos, Max Flach, Vicente Zamudio-Bayer, Michael Roemelt, J. Tobias Lau, Konstantin Hirsch

**Affiliations:** † Abteilung für Hochempfindliche Röntgenspektroskopie, Helmholtz-Zentrum Berlin für Materialien und Energie, Albert-Einstein-Straße 15, 12489 Berlin, Germany; ‡ Physikalisches Institut, Universität Freiburg, Hermann-Herder-Straße 3, 79104 Freiburg, Germany; § Institut für Chemie, Humboldt-Universität zu Berlin, Brook-Taylor-Straße 2, 12489 Berlin, Germany

## Abstract

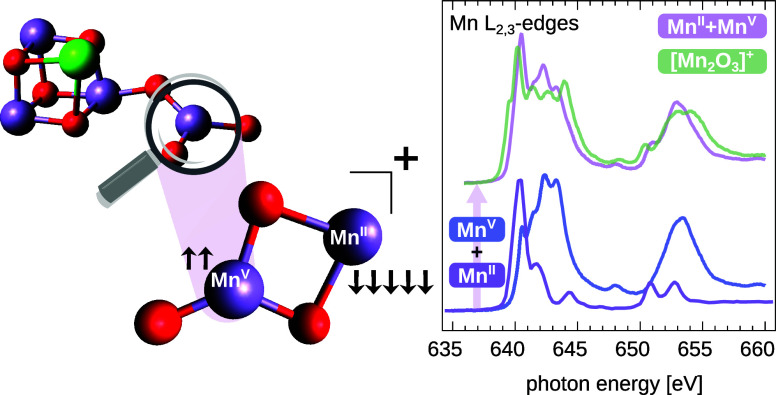

In a comprehensive investigation of the dinuclear [Mn_2_O_3_]^+^ cluster, the smallest dimanganese entity with two μ-oxo bridges and a terminal oxo ligand, and a simplified structural model of the active center in the oxygen-evolving complex, we identify antiferromagnetically coupled high-spin manganese centers in very different oxidation states of +2 and +5, but rule out the presence of a manganese(IV)-oxyl species by experimental X-ray absorption and X-ray magnetic circular dichroism spectroscopy combined with multireference calculations. This first identification of a high-spin manganese(V) center in any polynuclear oxidomanganese complex underscores the need for multireference computational methods to describe high-valent oxidomanganese species.

## Introduction

Manganese chemistry is complex and rich because of the large number of accessible oxidation states, and ranges from fundamental processes in water splitting and applications in water treatment to C–H bond activation.^1−5^ In particular, natural water splitting^1,6^ by an oxygen-bridged CaMn_4_O_5_ cubane cluster as the oxygen-evolving complex (OEC) of photosystem II points at an interesting problem: Although there is tremendous progress in understanding photosynthesis in the Kok cycle^7−9^ and despite the fact that the relevant state of OEC has been studied intensively, by serial femtosecond X-ray crystallography,^8,10−12^ reaction kinetics,^9,13^ electron paramagnetic resonance,^14−19^ and X-ray spectroscopy,^20−22^ the exact reaction mechanism that forms dioxygen is still under debate.^23,24^ At the heart of the problem of dioxygen formation is the nature of the active species that is described as one of the manganese(IV)-oxyl or manganese(V)-oxo valence tautomeric forms,^25−29^ which are linked to fundamentally different mechanisms of dioxygen formation, either by radical coupling^22,25^ or by nucleophilic oxygen–oxygen coupling.^27,30^ For lack of direct experimental data on the electronic structure of individual manganese centers in OEC, mechanistic interpretations often rely on density functional theory (DFT) calculations.^25,28,31,32^ These DFT studies predominantly favor manganese(IV)-oxyl species in OEC,^25,32,33^ whereas results on artificial water-splitting suggest manganese(V)-oxo species as crucial for dioxygen formation.^34−36^ As a further complication, the formation of dioxygen in its triplet ground state requires a local high-spin state of the active manganese center, but high-spin manganese(IV)-oxyl or high-spin manganese(V)-oxo species are elusive. Except for tetrahedrally coordinated manganese(V) in bulk brownmillerites and hypomanganates, which trivially form high-spin manganese(V)-oxo centers,^37,38^ only two experimentally verified high-spin oxidomanganese(V) complexes exist,^39−42^ both of which are mononuclear whereas natural oxygen evolution requires multinuclear complexes.^43^ Furthermore, no experimental evidence for any manganese(IV)-oxyl species has been found to date to the best of our knowledge. This problem has already been addressed in many experimental and computational studies on small polynuclear manganese model systems that contain bis(μ-oxo) bridged manganese centers as basic structural motifs of OEC. More than 100 of these species have been studied, all of which carry manganese centers in oxidation states of +2, + 3, or +4.^34,44^ Consequently, the question remains as to whether any manganese(V)-oxo or manganese(IV)-oxyl species can be identified in complexes with more than one manganese center.

Here we examine the [Mn_2_O_3_]^+^ cluster, the simplest bis(μ-oxo) bridged dimanganese complex with a terminal oxo ligand. We show, by X-ray absorption (XAS) and X-ray magnetic circular dichroism (XMCD) spectroscopy combined with multireference calculations, that [Mn_2_O_3_]^+^ in its ground state carries two strongly charge-disproportionated high-spin manganese centers in oxidation states of +2 and +5, respectively, while DFT consistently but incorrectly predicts a minimum energy structure of [Mn_2_O_3_]^+^ with the metal centers in oxidation states +3 and +4. Only a multireference approach is capable of correctly determining the electronic ground state. This might be relevant to any conclusion drawn on dioxygen formation by manganese centers from DFT calculations, and calls for further computational development to efficiently but correctly describe the processes in the oxygen-evolution reaction, where at least part of the multinuclear complex needs to be treated with a multireference approach.

## Results

### Electronic State of [Mn_2_O_3_]^+^ and Evidence for +2 and +5 Oxidation States of the Manganese Centers

**Figure 1:**
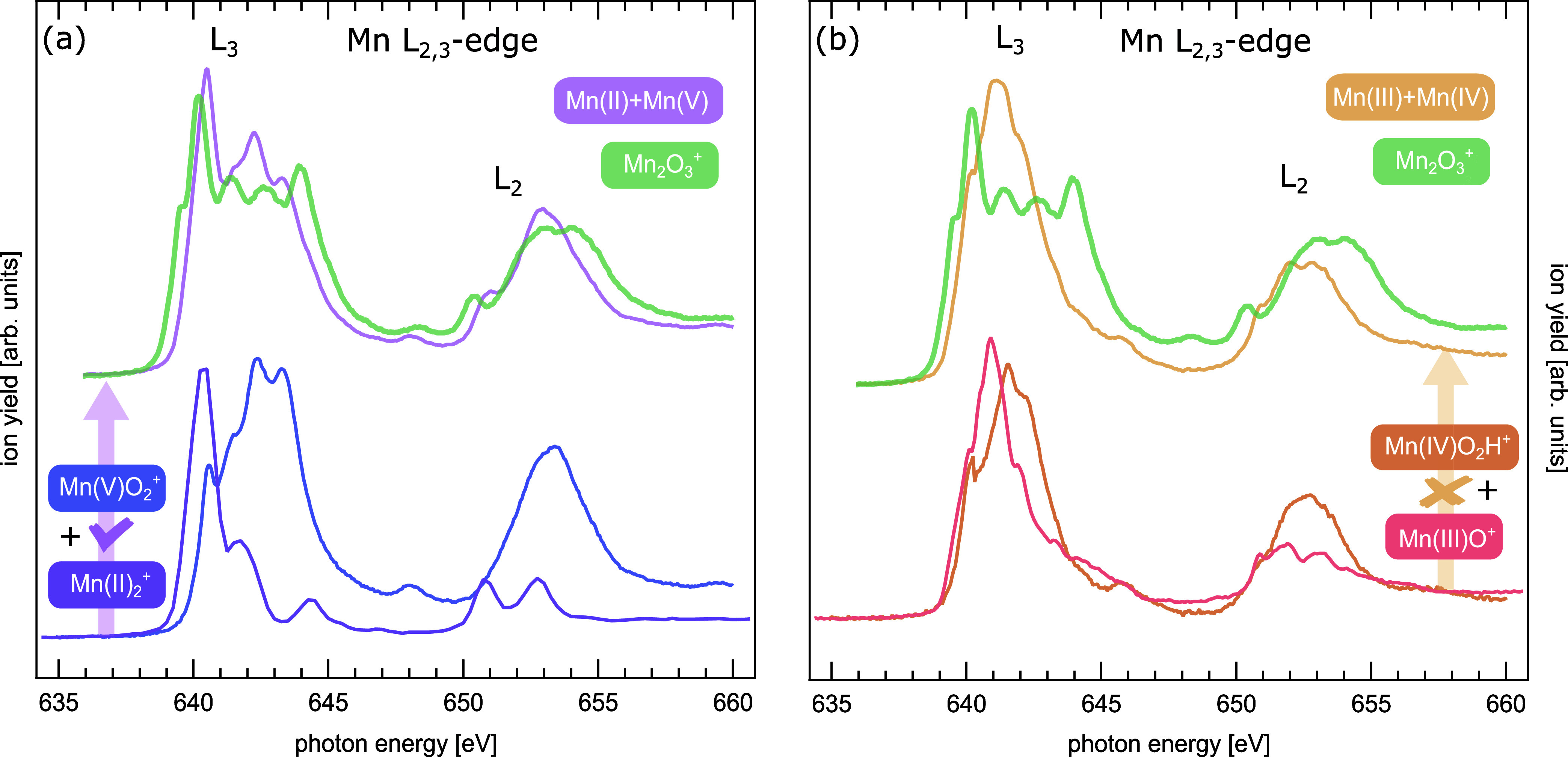
X-ray absorption spectra at the manganese L_2,3_ edges of [Mn_2_O_3_]^+^ (solid green trace), along with the fit of the sum of two reference spectra, [Mn_2_^II^]^+^ (purple trace)^45^ and [Mn^V^O_2_]^+^ (blue trace) in panel (a), and [Mn^III^O]^+^ (light red trace)^41^ and [Mn^IV^OOH]^+^ (orange trace)^42^ in panel (b). Only the combination of manganese centers in oxidation states +2 and +5 in panel (a) can reproduce the higher-energy transitions of [Mn_2_O_3_]^+^, in contrast to the centers in oxidation states +3 and +4 in panel (b). The fits of reference spectra to [Mn_2_O_3_]^+^ yield cosine similarities^46^ of 0.983 and 0.907 for (+2,+5) and (+3,+4), respectively.

Oxidation states can be derived from experimental L-edge X-ray absorption spectra either by determination of excitation-energy shifts,^47^ or by comparison to experimental^48^ or computational reference spectra, the latter often computed by wave function-based ab initio methods.^41,49−51^ In Figure 1, the X-ray absorption spectrum of [Mn_2_O_3_]^+^ at the manganese L_2,3_ edges is presented. Notably, the L_3_ edge is remarkably broad with a full-width at half-maximum (fwhm) of about 5.5 eV, but still shows well-resolved multiplet structure. The determination of median excitation-energy shifts (see SI for details) results in average oxidation states for multinuclear complexes, and yields +3.5 for [Mn_2_O_3_]^+^, which is compatible with three oxo-ligands, but not with an oxyl ligand. The reported structure of [Mn_2_O_3_]^+^, with its two oxygen-bridged manganese atoms and an additional terminal oxygen ligand, is compatible with suggested oxidation states +3 and +4 of the manganese centers, respectively.^52,53^ However, oxidation states of +2 and +5 would also be in agreement with an average oxidation state of +3.5 of the manganese centers in [Mn_2_O_3_]^+^. To determine the individual oxidation states of the two manganese centers of [Mn_2_O_3_]^+^, a more detailed analysis is required. Since reliable calculation of L-edge spectra, despite significant progress in the field of multireference approaches, is still limited to single transition-metal centers, because computational costs scale with the size of the active space,^54^ we here resort to experimental reference data, shown in Figure 1. This is justified because the L-edge of 3d transition-metal compounds is predominantly influenced by the occupation of the 3d-derived states,^55^ which is strongly interlinked with the oxidation state of the metal center.^56,57^ Additionally, oxo-ligands are weak ligands, positioned to the left in the spectrochemical series,^58^ typically resulting in small crystal fields. The anticipated effect of symmetry and crystal field on spectral shapes of the manganese centers in the reference systems for different oxidation states of manganese and [Mn_2_O_3_]^+^ should therefore be small.

By fitting reference spectra to the spectrum of [Mn_2_O_3_]^+^, cf. Figure 1, it becomes clear that a linear combination of spectra of manganese in oxidation states +3 and +4 cannot reproduce the spectrum of [Mn_2_O_3_]^+^, especially at the high-energy part of the L_3_ edge; whereas a combination of reference spectra of manganese in oxidation states +2 and +5 not only leads to better agreement at the high-energy tail of the L_3_ edge but also reproduces the overall structure of the L_3_ line considerably better. This is also evident from a quantitative analysis giving cosine similarities of 0.983 and 0.907 for the two combinations of oxidation states, respectively.^46^ See SI for details of the reference data and the fitting procedure.

The remaining small discrepancy in Figure 1(a) might result from the lower coordination of the manganese(V) center in [MnO_2_]^+^ as compared to [Mn_2_O_3_]^+^. We have recently shown that, within the same oxidation state of a manganese center, the L_3_ edge can shift by up to 0.44 eV per coordination number with increasing coordination.^49^ Moreover, even within the same oxidation state and coordination number, the median excitation energy can vary by up to 0.4 eV.^59^ Allowing for an energy shift of the manganese(II) and manganese(V) reference spectra in the fit, we achieve almost perfect agreement, reflected in a cosine similarity of 0.994, of the sum of the reference spectra of manganese in oxidation states +2 and +5 with the L-edge absorption spectrum of [Mn_2_O_3_]^+^, as can be seen from SI Figure 6. The resulting shift of the manganese(V) spectrum of 0.44 ± 0.04 eV is consistent with the lower coordination number of manganese in the reference spectrum. This is in stark contrast to the fit of a pair of reference spectra of manganese in oxidation states of +3 and +4, again with the absolute energy of the reference spectra as an additional free fit parameter, for which the best cosine similarity of 0.987 can only be obtained if the manganese(III) spectrum is shifted by −0.6 ± 0.08 eV, indicating a lower oxidation state than +3, and the manganese(IV) spectrum is shifted by 1.76 ± 0.04 eV, indicating a higher oxidation state than +4, see SI Figure 7. Clearly, the resulting relative energy shift of 2.36 ± 0.09 eV contradicts the initial assumption that oxidation states would only differ by one, but indicates again that oxidation states of both manganese centers differ by three, compatible only with manganese oxidation states of +2 and +5 in [Mn_2_O_3_]^+^.

Our experimentally assigned oxidation states are corroborated by our theoretical findings. The electronic ground state of [Mn_2_O_3_]^+^ is ^4^A_2_ (C_2*v*_) at the DMRG-NEVPT2 level,^60,61^ see Table 1 for relative energies, and SI section 4 for a detailed discussion of the computational procedure. Analysis of the occupation of the localized DMRGSCF orbitals (see SI Figures 10–13) yields manganese oxidation states +2 and +5 in agreement with Mulliken spin populations, known to be a reliable measure of oxidation states in manganese-oxo systems,^62^ at the manganese centers of the same ^4^A_2_ (C_2*v*_) state at the DMRG/HCI^63^ (4.1 and −1.2) and DFT (4.64 and −2.02) level. Hence, we clearly demonstrate manganese centers in oxidation states +5 and +2 in dinuclear [Mn_2_O_3_]^+^, both experimentally and at the DMRG-NEVPT2 level. The ^4^A_2_ (C_2*v*_) global minimum is more stable by 260 meV than the lowest-energy structure with oxidation states of +3 and +4 of the manganese centers. In contrast, DFT consistently seems to predict the wrong energetic order of isomers, and prefers oxidation states of +3 and +4 in our and other studies.^52,53^ For more details on the energetics of the isomers at different levels of theory see SI section S4.

**Table 1:** Total Spin Multiplicity, Individual Oxidation States, Representation of the Local Spins to Illustrate Interatomic Spin Coupling, and Relative Energies at DFT and DMRGSCF/SC-NEVPT2 Levels of Low-Energy Species of [Mn_2_O_3_]^+,^^a^

2S + 1	oxidation states	spin states	relative energy [eV]	
	(Mn, Mn)	(O, Mn, Mn)	DMRG-NEVPT2	TPSSh
4	(+5,+2)	(↑↓, ↑↑, ↓↓↓↓↓)	0	0.10
6	(+5,+2)	(↑↓, ↑↓, ↓↓↓↓↓)	0.06	0.37
8	(+5,+2)	(↑↓, ↑↑, ↑↑↑↑↑)	0.22	0.24
2	(+4,+3)	(↑↓, ↑↑↑, ↓↓↓↓)	0.26	0
8	(+4,+3)	(↑↓, ↑↑↑, ↑↑↑↑)	0.39	0.13
8	(+4,+2)	(↓, ↑↑↑, ↑↑↑↑↑)		0.82
10	(+4,+2)	(↑, ↑↑↑, ↑↑↑↑↑)		1.40

a While in DFT (+2,+5) and (+3,+4) isomers are very close in energy, the three energetically lowest isomers at DMRGSCF/SC-NEVPT2 level are all (+2,+5), and the (+2,+5) ground state is more stable by 260 meV than the lowest (+3,+4) isomer. States where a manganese(IV) center is bound to an oxyl radical are very high in energy, at least 0.82 eV above the global minimum in DFT. These high-energy manganese(IV) oxyl isomers did not convergence at the DMRGSCF level.

### Excluding Possible Oxyl-Character of [Mn_2_O_3_]^+^

**Figure 2:**
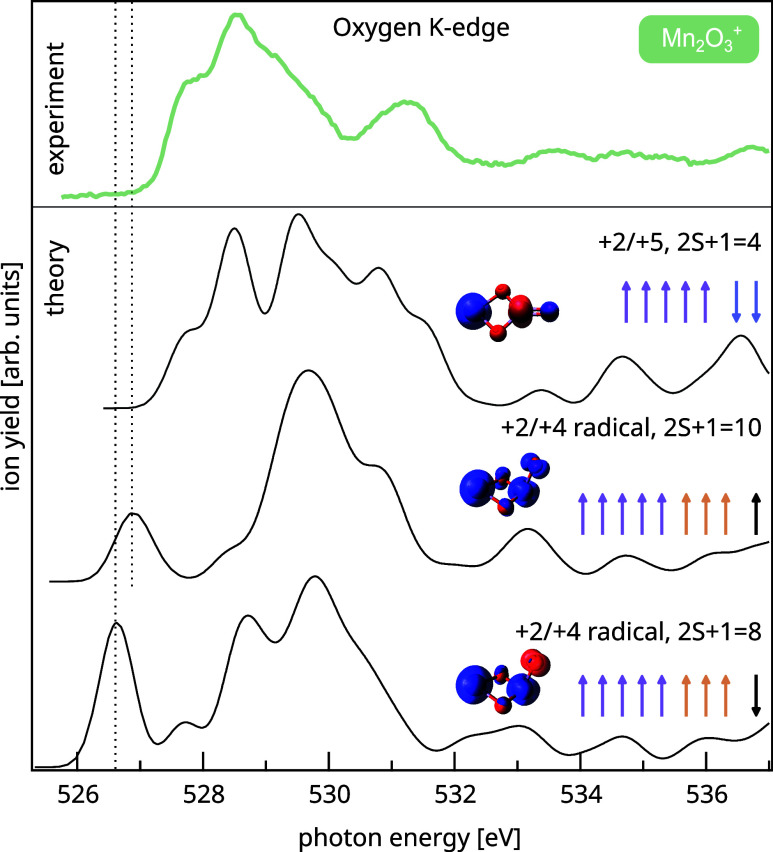
Experimental oxygen K-edge spectrum of [Mn_2_O_3_]^+^ compared to the calculated TD-DFT spectra of the ^4^A_2_ (C_2*v*_) ground state of [Mn_2_O_3_]^+^ as identified by DMRG-NEVPT2, and to selected excited states of [Mn_2_O_3_]^+^ with oxyl radical character. All calculated spectra are shifted by +13.3 eV to match the experimental spectrum. The calculated spectrum of the global minimum species matches the experimental spectrum best. The low-energy transition around 526.5 eV, indicated by dashed lines and present only in the calculated spectra of isomers with manganese(IV)-oxyl-character, is a signature of an oxygen-centered radical.^65,66^ The absence of this transition in the experimental spectrum rules out any contribution of an oxyl species in [Mn_2_O_3_]^+^. Additionally shown are spin densities for each species. Owing to covalent bonding, there is a finite spin population at the terminal oxygen of 0.37 in the ground state. This value is, however, significantly smaller than the spin population of 0.93 in the two manganese(IV)-oxyl species.

To further corroborate our compelling experimental and theoretical evidence for manganese centers of oxidation states +2 and +5 in [Mn_2_O_3_]^+^, we carried out XAS at the oxygen K-edge to probe any possible radical character of the oxo-ligands that might result from small or inverted ligand fields.^64^ Experimentally, the presence of an oxygen-centered radical can be tested by a distinct low-energy transition at about 526.5 eV in oxygen K-edge X-ray absorption.^65,66^ Our X-ray absorption spectrum at the oxygen K-edge of [Mn_2_O_3_]^+^ is depicted in Figure 2. The experimental data is well reproduced by the TD-DFT calculated spectrum for the ^4^A_2_ (C_2*v*_) ground state. The agreement is particularly good at low excitation energies, while the high-energy transitions are known to be less well reproduced due to approximations employed in TD-DFT.^67^ Importantly, neither the experimental nor the computational spectrum display the typical low-energy excitation associated with an oxygen-centered p-hole at about 526.5 eV.^65,66^ Instead, the lowest-energy excitation is a transition into a manganese–oxygen hybrid molecular orbital of strong manganese character with significant admixture of p-orbitals from the terminal oxygen atom. In contrast to the calculated oxygen K-edge spectrum of the lowest-energy manganese(V)-oxo species, the computed spectra of the two manganese(IV)-oxyl isomers of [Mn_2_O_3_]^+^ exhibit the characteristic excitation at lower photon energies, as expected (see Figure 2). Even more, the lowest-energy oxyl radical species of [Mn_2_O_3_]^+^ is at 0.82 eV at the DFT level as listed in Table 1, and could not be located at all at the DMRG-NEVPT2 level. Thus, an oxygen-centered radical in the ground state of [Mn_2_O_3_]^+^ can be ruled out from our experimental and theoretical data. Still, the strong hybridization of manganese and oxygen orbitals results in a sizable spin density at the terminal oxygen atom even for the ^4^A_2_ (C_2*v*_) manganese(V)-oxo species as can be seen from the spin densities shown in Figure 2.

In order to further elaborate on this, we quantify oxygen spin populations as well as manganese–oxygen bond lengths in the state-specifically optimized structures. We find a manganese–terminal-oxygen bond length of 1.54 Å for [Mn_2_O_3_]^+^ in its ground state, in good agreement with the range of bond lengths of 1.55–1.59 Å reported for manganese(V)-oxo species, but in contrast to the expanded bond length of 1.76 Å reported for manganese(IV)-oxyl entities,^41,68−72^ which again agrees with 1.72 Å for our manganese(IV)-oxyl isomers of [Mn_2_O_3_]^+^ presented in Figure 2. Because small molecular systems like [Mn_2_O_3_]^+^ are covalently bound, a clear assignment of spin densities to either one of the extreme cases, manganese(V)-oxo or manganese(IV)-oxyl, is less obvious, as evident from the nonvanishing spin density at the terminal oxygen ligand, and from the manganese–oxygen hybrid character of the LUMO. Consequently, reported spin populations at the oxygen ligand span a much wider range than bond distances. For manganese(IV)-oxyl species, reported spin populations range from 0.43^27^ to 0.85,^68^ while they range from 0.14^27^ to 0.45^40^ for manganese(V)-oxo species. Here, we find a spin density at the terminal oxygen atom of 0.37 for the ^4^A_2_ (C_2*v*_) ground state of [Mn_2_O_3_]^+^, in agreement with the range given for terminal oxo-ligands but below the range given for oxyl species. Thus, combining our experimental and computational evidence, the presence of an oxyl ligand in [Mn_2_O_3_]^+^ can be safely ruled out.

### Antiferromagnetic Coupling of Local High-Spin States at the Manganese Centers

**Figure 3:**
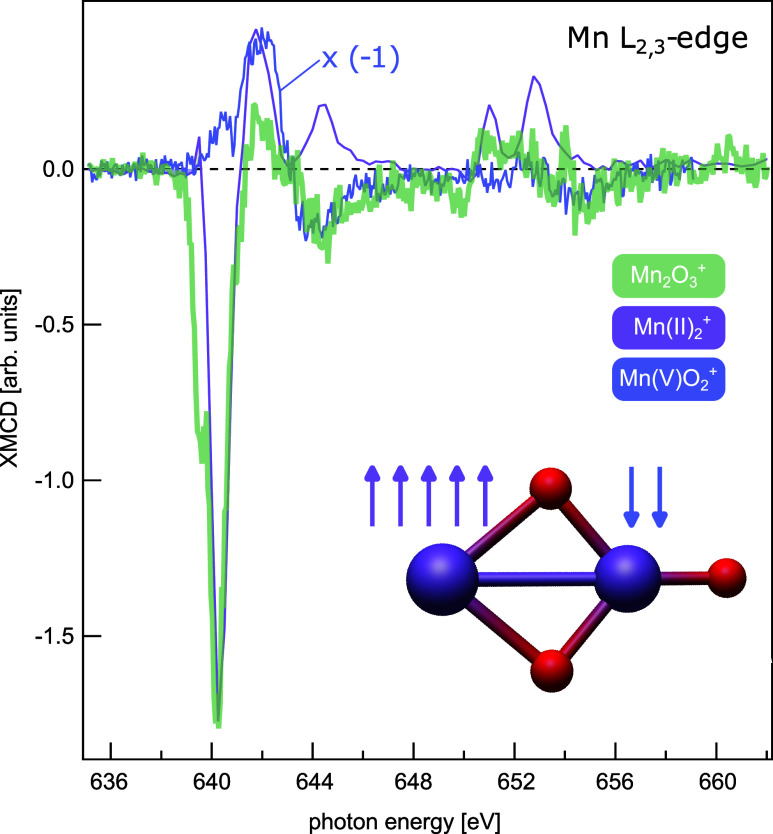
Experimental XMCD spectrum of [Mn_2_O_3_]^+^ (green trace), compared to XMCD reference data of manganese(II) in [Mn_2_]^+^^45^ (purple trace) with its dominant, negative contribution at 640 eV, and of manganese(V) in [MnO_2_]^+^ (blue trace).^42^ The XMCD pattern of [Mn_2_O_3_]^+^ at 640 eV agrees with manganese(II), but follows the data, inverted in sign, of manganese(V) above 643 eV. This indicates antiferromagnetic coupling of the local high-spin states at the manganese(II) and manganese(V) centers in [Mn_2_O_3_]^+^, leading to an overall quartet spin state.

The XMCD spectrum of [Mn_2_O_3_]^+^, displayed in Figure 3, shows a clear dichroism, indicative of a net magnetic moment. Since the XMCD spin sum rule breaks down, and prohibits quantitative analysis for early transition metals, or for transition metals in high oxidation states,^73^ XMCD reference spectra of high-spin manganese(II) in [Mn_2_]^+^,^45^ and of high-spin manganese(V) in [MnO_2_]^+^^42^ are used to identify local high-spin states, and their coupling, in XMCD of [Mn_2_O_3_]^+^ that, in zero order approximation for two localized magnetic moments at the manganese centers, should be a linear superposition of the reference XMCD signals. For details of the spectral analysis see SI.

Most prominent in the XMCD signal of [Mn_2_O_3_]^+^, cf. Figure 3, is the significant, negative intensity around 640 eV, which is reproduced by the reference spectrum of manganese(II), and can thus be attributed to the local high-spin manganese(II) center of [Mn_2_O_3_]^+^. The vanishing contribution of manganese(II) to the higher-energy part, above 642 eV, of the XMCD spectrum can be reproduced by variation of the Slater integrals in Hartree–Fock calculations,^74^ and is explained by covalent bonding of manganese(II) to the bridging oxygen, absent from the reference spectrum. Above 642 eV, the XMCD signal of [Mn_2_O_3_]^+^ follows the sign-inverted XMCD of high-spin manganese(V), except for a deviation around 650 eV, where a remaining manganese(II) contribution is visible, see SI for details. The good agreement with the model spectra allows us to also assign a local high-spin manganese(V) center, and to conclude antiferromagnetic coupling of both centers, in agreement with the ^4^A_2_ (C_2*v*_) ground state predicted by DMRG-NEVPT2 theory, cf. Table 1. Interestingly, for all models of the state S_4_ of the Kok cycle that are currently considered, the spins of manganese atoms involved in O_2_ formation also couple antiferromagnetically, which is a prerequisite to form dioxygen in a triplet state.^24,27,30,75,76^

In addition to the ^4^A_2_ (C_2*v*_) ground state, our DMRG-NEVPT2 calculations also predict a close-lying sextet state, with a local low-spin state at the manganese(V) center, at only 0.06 eV relative energy, below the accuracy of the theory, and therefore considered degenerate. Although a minor population of [Mn_2_O_3_]^+^ in this sextet state cannot be ruled out from the experimental data, we exclude a dominant contribution because of the good agreement with the local high-spin state of manganese(V) in contrast to the vanishing XMCD signal of a singlet manganese(V). This also indicates that the sextet state is indeed higher in energy than the quartet state. This close-lying local low-spin state might, however, offer a spin degree of freedom that could be beneficial in chemical reactions with specific requirements for the spin states of the products, such as triplet dioxygen formation.

### Discussion

As concluded above, [Mn_2_O_3_]^+^, as a simple bis(μ-oxo) bridged dinuclear manganese complex with a terminal oxo ligand, is the first reported dimanganese-oxo species to exhibit a high-spin manganese center in oxidation state +5. Furthermore, this high-spin manganese(V) center is coupled antiferromagnetically, via bis(μ-oxo) bridges, to a high-spin manganese(II) center, resulting in a coordination entity that sustains two strongly charge-disproportionated manganese centers in very different oxidation states of +2 and +5.

Our finding of high-spin manganese(V) is in line with artificial water splitting, where manganese(V)-oxo species are proposed to play an essential role.^34−36^ In natural water splitting, the mechanism of dioxygen formation is still unknown,^12,23,24^ and whether manganese(IV)^22,25^ or manganese(V)^27,30^ centers, in combination with oxo or oxyl ligands, are formed, is an open question. For [Mn_2_O_3_]^+^ our results clearly indicate a high-spin manganese(V)-oxo center in the lowest-energy species but exclude the presence of manganese(IV)-oxyl, although strongly covalent bonding between the manganese center and the terminal oxygen inevitably leads to spin density at the oxygen site and blurs the clear distinction between oxo and oxyl species in computational approaches. This has already been hinted at for [Mn^V^H_3_buea(O)]^40,77^ but, as shown here, also holds true for polynuclear manganese-oxo species.

Our experimental and computational evidence shows that oxidation of the manganese center is preferred over oxyl formation even if this leads to strong charge disproportionation in [Mn_2_O_3_]^+^. This finding challenges results based on DFT, which favors a manganese(IV)-oxyl species as the active site of the OEC, but for which spin-energetics and the amount of radical character strongly depend on the choice of functional.^70,78,79^ Even for the simplest bis(μ-oxo) bridged dimanganese unit with a terminal oxygen ligand, popular density functionals do not describe the lowest-energy species, or energetic ordering of [Mn_2_O_3_]^+^ isomers, correctly, neither in our nor in other studies.^52,53^ Instead, multireference methods, DMRG-NEVPT2 in our case, need to be employed to find the correct energetics for [Mn_2_O_3_]^+^. Hence, DFT-based predictions do not describe the energetics of transition-metal oxides correctly, in particular those of high-valence states, but might erroneously favor a + 4 oxidation state and oxyl ligand, e.g., as in the case of the active center in OEC. In light of our findings, any proposed mechanism of dioxygen formation at high-valent manganese centers that is solely based on DFT computations without direct experimental evidence of the electronic structure should be viewed with caution even if the calculations are seemingly consistent with experimental structural data.^80^

Although the lowest-energy structure of [Mn_2_O_3_]^+^ exhibits structural similarity to the proposed dangling manganese(V) site of OEC, the manganese(V) center in [Mn_2_O_3_]^+^ possesses lower, i.e., 3-fold, coordination (see SI for details) and lacks any ligands or substrate water molecules. It would therefore be interesting to study model systems with 6-fold-coordinated manganese centers that are structurally closer to the proposed manganese(IV)-oxyl species, or to study model systems with 5-fold-coordinated manganese centers as even better structural models of the proposed manganese(V) active site in OEC than [Mn_2_O_3_]^+^. These experiments could elucidate whether a change in symmetry could indeed induce an inverted ligand field that would result in localization of the spin density at the oxygen site.^27,32^ Such an approach would also facilitate further investigation into the reliability of DFT in predicting the oxidation states of manganese centers in more complex ligand environments; and could indicate whether our [Mn_2_O_3_]^+^ model system represents an unusual exception or whether high-spin manganese(V) centers in polynuclear manganese-oxo complexes might be more common than expected.

In summary, our results clearly demonstrate that elusive high-spin manganese(V) exists in the ground state of a dimanganese oxide complex, while no experimental observation of any manganese(IV)-oxyl species has been reported yet, despite numerous theoretical predictions.^25,81^ Although any conclusion on our [Mn_2_O_3_]^+^ model complex can only be transferred carefully to OEC, it still underlines the need to reassess computational models that are based on DFT only. In this respect, our findings might advance the understanding of possible oxidation and spin states in polynuclear oxidomanganese complexes of relevance not only to biological and artificial water splitting, but also to high-valent manganese redox chemistry in general.

## Methods

### Experimental Details

The XAS and XMCD measurements were carried out at the ion trap endstation,^82,83^ located at beamline UE52-PGM of the synchrotron radiation facility BESSY II operated by Helmholtz-Zentrum Berlin.

Dimanganese oxide cations are produced by DC-sputtering of a manganese target of 99.95% purity in a helium and argon atmosphere at liquid nitrogen temperature while simultaneously introducing trace amounts of oxygen to the discharge. The ion beam is then guided via a hexapole ion guide and quadrupole mass filter, selecting the species of interest, to a liquid-helium cooled quadrupole ion trap for cooling of the clusters to a temperature of approximately 20 K. Typical mass spectra are shown in SI Figure 1. Photon energy calibration was performed using the neon 1s excitation in the beamline ionization cell and verified at the oxygen K-edge, giving a photon energy uncertainty of ±0.1 eV. X-ray absorption spectra at the oxygen K-edge were recorded using a linearly polarized X-ray beam by tuning the photon energy across the oxygen K-edge between 520 and 540 eV. The spectra were recorded with a step width of 50 meV and bandwidth of 128 meV. XAS was performed in ion yield mode which, at the absorption edges of interest, is a good approximation of the X-ray absorption cross section. The parent and product ions were detected by a reflectron time-of-flight mass spectrometer. A compilation of the fragmentation channels and partial in yield spectra for different product ions is given in SI section 2. For the XMCD measurements, a superconducting solenoid creates a homogeneous magnetic field of μ_B_H = 4.95 T along the trap axis to magnetize the sample. Ion yield spectroscopy is performed by collecting product ions resulting from X-ray absorption of photons with helicity parallel and antiparallel to the magnetic field axis, respectively.^84^ The photon energy was tuned across the manganese L_2,3_ edges from 630 to 670 eV, with a bandwidth of 167 meV and a step width of 60 meV. Partial ion yield spectra are presented in SI Section 2 for showcasing the stability and reproducibility that is necessary for XMCD spectroscopy. The XAS signal was derived from an average of X-ray absorption spectra recorded with opposite circular polarization.

Furthermore, we can exclude two major sources of radiation damage to the sample. Commonly, the radiation damage-induced reduction of a sample is caused by the Auger electrons of a support or solvent. In contrast, in our gas-phase experiments, the only source of Auger electrons, apart from the sample itself, is the helium buffer gas. However, because of the low absorption cross section, below 5 × 10^–3^ Mbarn,^85^ of helium at the manganese L-edge and oxygen K-edge, and because of the low number density of helium of typically 10^14^ atoms cm^–3^, orders of magnitude below condensed matter in solution or deposited samples, any reduction of our sample by radiation damage is highly unlikely. Furthermore, we estimate the upper limit for the contribution of sequential two-photon X-ray absorption to the X-ray absorption spectra, which would lead to additional ionization or dissociation, to be 10^–3^.^82^

### Computational Details

This work encompasses reports of quantum chemical calculations on different levels of theory using multiple programs. In the following, technical details of the different sets of calculations are described.

All DFT calculations presented in this work were conducted with the ORCA program package in its version 5.0.3.^86^ As outlined in the scientific sections of this manuscript, molecular geometries were optimized for various electronic states within the framework of broken-symmetry density functional theory (BSDFT). Based on the success in previous studies,^87−89^ the TPSSh functional^90,91^ was utilized for this step of the computational studies. The main basis set used was def2-TZVP,^92^ whereas the def2/J auxiliary basis set was used during the Coulomb matrix builds with the density fitting.^93−95^ For treating the Hartree–Fock exchange, the COSX approximation was used.^96,97^ Additionally, the D3 dispersion correction with the Becke–Johnson damping was enabled.^98^

Oxygen K-edge XAS was simulated by means of time-dependent density functional theory calculations employing the TPSSh functional, while allowing for the excitation of electrons from molecular orbitals with a primary ligand 1s character.^99^ Oxygen 1s orbitals were localized using the Pipek–Mezey scheme to simulate the underlying phenomena.^100^ The Tamm–Dancoff approximation^101^ was employed in all TD-DFT calculations, and scalar relativistic effects were approximated using the zeroth-order regular approximation (ZORA).^102,103^ Simulated spectra were generated by convoluting the calculated transitions with Gaussian functions of 0.65 eV width to simulate both experimental resolution and lifetime broadening.^49^ All computed spectra were shifted by +13.3 eV to align with experimental spectrum, a necessary adjustment to account for systematic errors introduced by the TD-DFT method and dependent on the chosen functional and basis set.^99^

Additional calculations that used wave function-based multireference (MR) electronic structure methods were conducted to obtain refined single point energies of the different [Mn_2_O_3_]^+^ isomers with different spin and oxidation state distributions. All reported MR calculations were performed with the HUMMR program, formerly named MOLBLOCK.^61^ The def2-TZVP basis set was employed for all atoms. All required two-electron integrals were evaluated utilizing the density-fitting approximation with the def2-TZVP-C basis set. The ASS1ST scheme was used to select a suitable active orbital space of (25e, 20o) and generate suitable starting orbitals.^110^ Since this active space size is out of reach for conventional, Full-CI based CASSCF calculations, the density matrix renormalization group implementation in the BLOCK program was used as approximate solver^54^ for the active space Full-CI equations.^104−106^ During these DMRGSCF calculations, a bond dimension of *m* = 500 was employed leading to the overall largest discarded weight of 1.332 × 10^–15^ for the octet. After DMRGSCF calculations were brought to convergence with the super-CI approach^107^ dynamical correlation effects were taken into account through second order *n*-electron valence state perturbation theory (NEVPT2) in its strongly contracted form.^60,108^ During these calculations a reverse schedule^109^ was employed with a maximal bond dimension of *m* = 1000 and a final bond dimension of *m* = 600. Active space spin densities and spin populations were subsequently obtained from configuration-based heatbath-CI calculations^63^ utilizing the converged DMRGSCF orbitals.

## Supporting Information Available

The following files are available free of charge. The Supporting Information is available free of charge at https://pubs.acs.org/doi/10.1021/jacs.4c14543.

Additional experimental data such as mass spectra, different ion yield channels, Hartree–Fock simulations of the XMCD signal, additional computational details including coordinates of all low lying isomers of [Mn_2_O_3_]^+^ (PDF)
