# Supplementary material for: High-Spin Manganese(V) in an Active Center Analogue of the Oxygen-Evolving Complex
Source: J Am Chem Soc. 2025 Feb 19;147(9):7336–44. doi: 10.1021/jacs.4c14543 (PMC11887058; doi:10.1021/jacs.4c14543)
Supplement: Supplementary file 1 — ja4c14543_si_001.pdf [file ja4c14543_si_001.pdf]

Supplementary Information for High-Spin Manganese(V) in an Active Center Analogue of the Oxygen-Evolving Complex

Olesya S.Ablyasova[1,2], Mihkel Ugandi[3], Esma B. Boydas[3], Mayara da Silva Santos[1,2], Max Flach[1,2], Vicente Zamudio-Bayer[1], Michael Römelt[3], J. Tobias Lau[1,2], and Konstantin Hirsch[1]

[1]Abteilung für Hochempfindliche Röntgenspektroskopie, Helmholtz-Zentrum Berlin für Materialien und Energie, Albert-Einstein-Straße 15, 12489 Berlin, Germany
[2]Physikalisches Institut, Universität Freiburg, Hermann-Herder-Straße 3, 79104 Freiburg, Germany
[3]Institut für Chemie, Humboldt-Universität zu Berlin, Brook-Taylor-Straße 2, 12489 Berlin, Germany

Contents

S1 Cluster Production and Mass Spectrometry **S3**

S2 Partial Ion Yield Spectra **S4**

S3 Comparison to Reference Spectra **S6**

S3.1 Average Oxidation State S7

S3.2 Quantitative Analysis of Spectral Shapes in XAS S8

S3.3 Detailed Discussion of the XMCD Signal and Spin State of $[Mn_2O_3]^+$ S10

S3.4 Influence of Hybridization on the XMCD Signature of Manganese(II) S12

S4 Computational Procedure to Identify Geometrical and Electronic Ground State of $[Mn_2O_3]^+$ **S13**

S4.1 Coordinates of $[Mn_2O_3]^+$ Isomers and Selected Structural Parameters of $[Mn_2O_3]^+$ and S_4^B S15

S4.2 Comparison of the Electronic Structure of the Manganese(V) center in $[Mn_2O_3]^+$ and the Dangling Manganese in S_4^B S18

S5 Localized DMRGSCF Orbitals **S19**

S1 Cluster Production and Mass Spectrometry

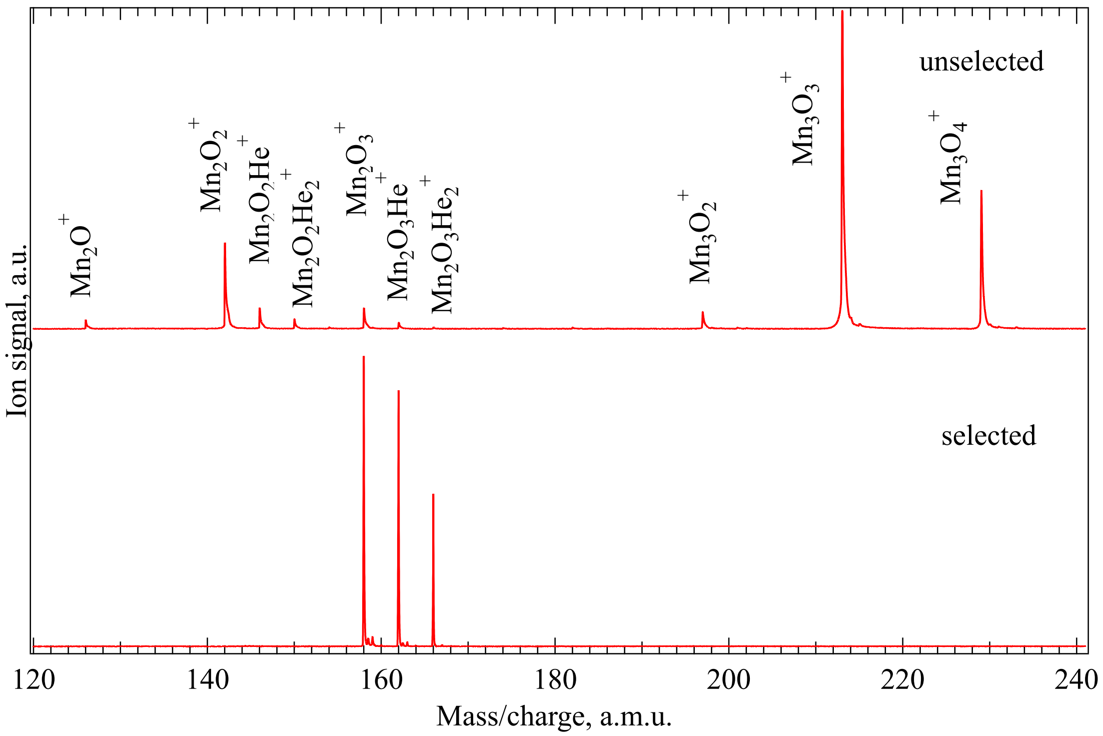

SI Figure 1: Mass spectrum of $[Mn_{2,3}O_{1-4}]^+$ as produced by the magnetron sputtering cluster source (upper panel). The lower panel shows a typical mass spectrum of mass selected $[Mn_2O_3]^+$. When storing the parent cluster in the ion trap filled with helium buffer gas for thermalization at cryogenic temperatures of about 20 K, attachment of several helium atoms can occur as can be seen in the lower panel.

As can be seen in the mass spectrum presented in SI figure 1, manganese oxide clusters of different composition can be produced. The preferred production of stoichiometric manganese oxide clusters that is also observed here has been reported before also for other cluster sources. [1, 2]

S2 Partial Ion Yield Spectra

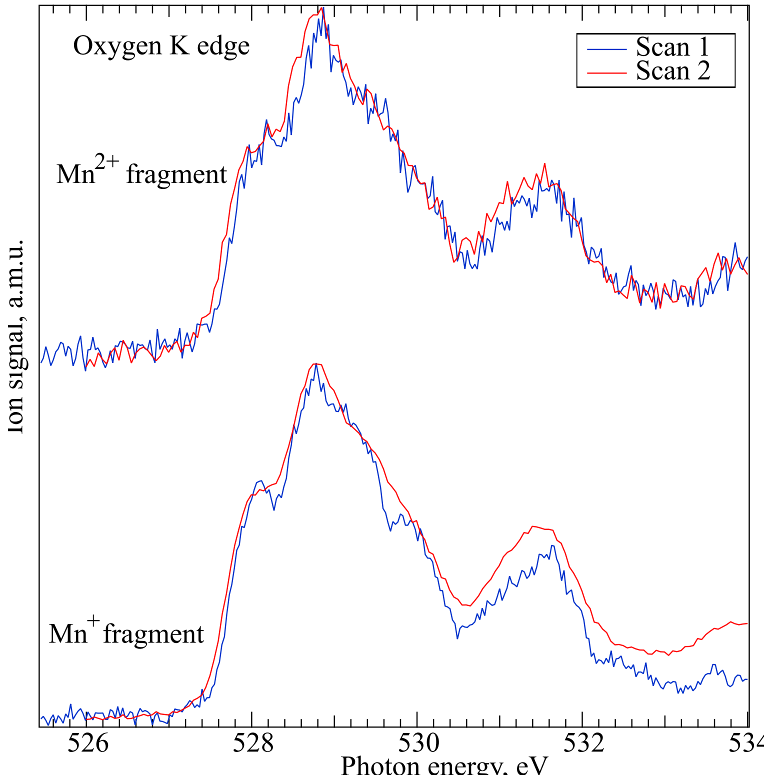

SI Figure 2: Averaged oxygen K-edge absorption spectra of $[Mn_2O_3]^+$ for different ion yield channels as measured during two different beamtimes (Scan 1 and Scan 2). The different ion yield channels show the same energy dependence. Therefore the measured partial ion yield can be assumed to be a good measure of the x-ray absorption.

X-ray absorption results in photofragmentation of the parent cluster $[Mn_2O_3]^+$. While the total ion yield at these excitation energies is proportional to the x-ray absorption, the energy dependence of the partial ion yield can in principle vary for the various product ions. In SI table 1 we list all the observed product ions at both the oxygen K- and manganese $L_{2,3}$ edges. Additionally, we show the energy dependence of the partial ion yield for all the fragments observed at both edges in SI figures 2–3. As can be seen, the partial ion yield at the oxygen K-edge does not depend on the photofragmentation channel and can therefore be considered to

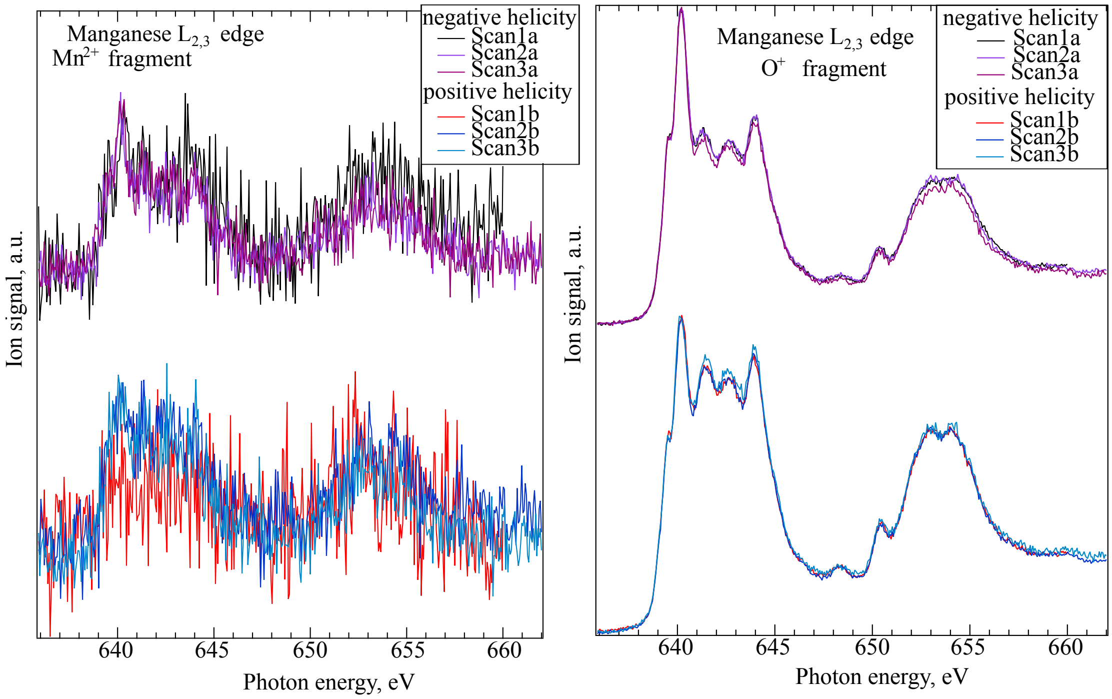

SI Figure 3: Partial ion yield spectra of $[Mn_2O_3]^+$ at the manganese $L_{2,3}$ edges measured with positive and negative helicity by switching the polarization of the x-ray beam. Shown is the ion yield spectra of the O^+ (right side) and Mn^{2+} (left side) product ions.

be proportional to the x-ray absorption. Moreover, at the manganese $L_{2,3}$ edges we observe only one intense product ion, O^+. Therefore this partial ion yield spectrum can be considered a good measure for the X-ray absorption spectrum. Furthermore, we show in SI figure 2 the oxygen K-edge absorption measured in two different beamtimes, highlighting the reproducibility of the data. In SI figure 3 we display the individual scans that also show the degree of reproducibility that is essential to record a XMCD spectra.

SI Table 1: Experimentally observed product ions of the $[Mn_2O_3]^+$ parent cluster at the oxygen K and manganese $L_{2,3}$ edges.

oxygen K edge	manganese $L_{2,3}$ edges
Mn^{2+}, Mn^+	Mn^{2+}, O^+

S3 Comparison to Reference Spectra

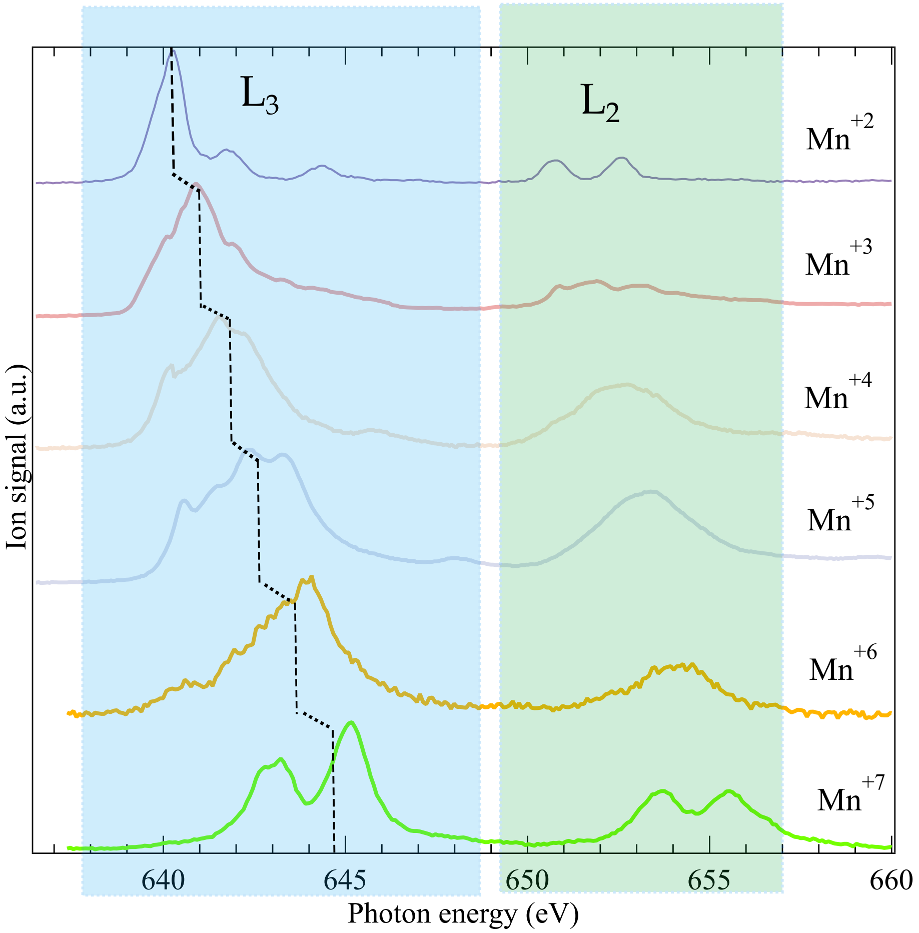

SI Figure 4: Compilation of experimental X-ray absorption spectra at the manganese $L_{2,3}$ edges of manganese oxides in oxidation states +2 through +7 used as reference spectra throughout this work. From top to bottom: X-ray absorption spectrum of $[Mn^{II}_2]^+$ [3], $[Mn^{III}O]^+$ [4], $[Mn^{IV}O_2H]^+$ [5], $[Mn^{V}O_2]^+$ [4, 5], $[Mn^{VI}O_4]^+$ [4], $[Mn^{VII}O_3]^+$ [4]. The median excitation energy of the L_3 edge is indicated by the dashed black line.

For reference spectra of manganese in its oxidation states of +2 to +5, we rely on $[Mn^{II}_2]^+$ [3], $[Mn^{III}O]^+$ [4], $[Mn^{IV}O_2H]^+$ and $[Mn^{V}O_2]^+$ [5], respectively. These are shown for comparison alongside $[Mn^{VI}O_4]^+$ and $[Mn^{VII}O_3]^+$ in SI figure 4.

S3.1 Average Oxidation State

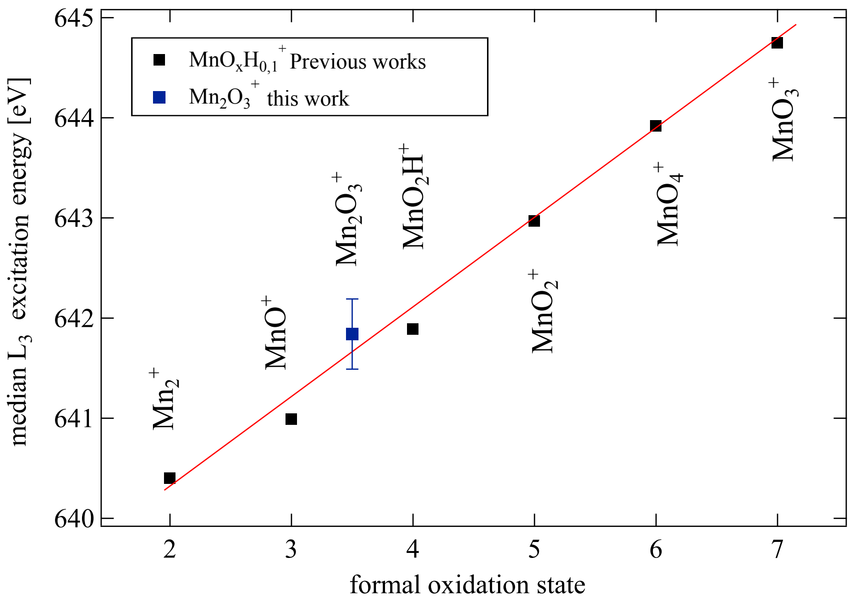

SI Figure 5: Experimental manganese L_3 median excitation energy as a function of the formal oxidation state for a series of manganese oxides adopting oxidation states +2 through +7 as reported in [3, 4, 5] (black dots). Additionally, the L_3 median excitation energy for $[Mn_2O_3]^+$ (blue square) is shown at an average oxidation state of +3.5 which holds irrespective of whether the manganese centers are in oxidation +3 and +4 or +2 and +5, respectively, but would be +3 if a manganese(IV)-oxyl species were present. The red line is a linear fit ($E_{median}(L_3) = a + b \cdot (\text{formal oxidation state})$) to the data without the $[Mn_2O_3]^+$ data point. The resulting L_3 median shift per oxidation state is $b = 0.90 \pm 0.09$ eV (and $a = 638.41 \pm 0.45$ eV).

As discussed in the main text, oxidation states are often inferred from median L_3 edge excitation energies. Since there is a linear relation between L_3 median excitation energy and the metal oxidation state [6, 7], for $[Mn_2O_3]^+$ this should result in a median L_3 excitation energy that corresponds to an average oxidation state of +3.5, when the manganese centers are in oxidation states +3 and +4 or +2 and +5, respectively. However, in case of the presence of an manganese(IV)-oxyl center the median L_3 excitation energy should rather correspond to that of an average oxidation state of 3. Therefore, we compiled this data for a series of

manganese oxides [4, 5] as presented in SI figure 5. Additionally, we show the median L_3 excitation energy of $[Mn_2O_3]^+$, which fits an average oxidation state of +3.5 within the error bars but does not fit an oxidation state of +3. This is a strong indication that no manganese(IV)-oxyl species is present.

S3.2 Quantitative Analysis of Spectral Shapes in XAS

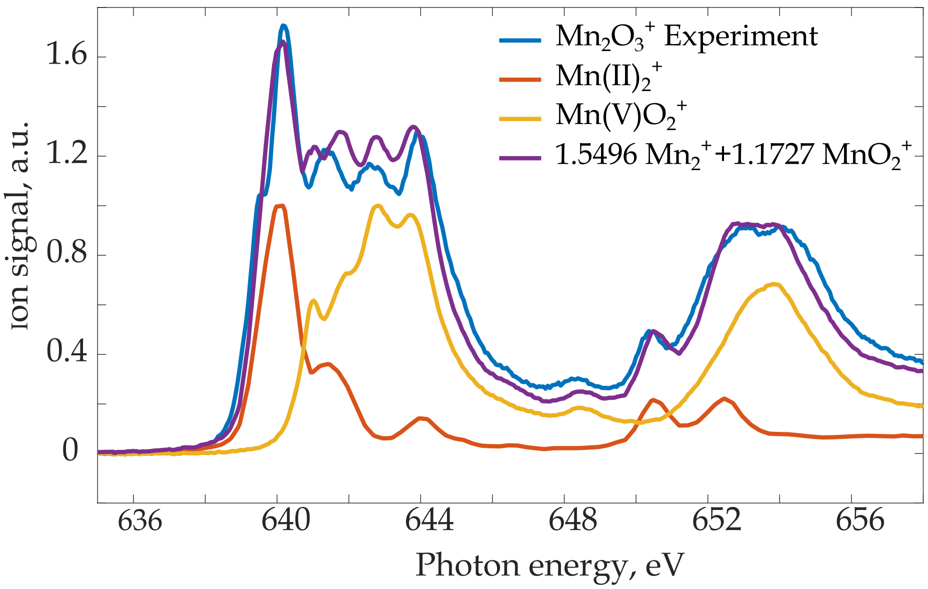

SI Figure 6: Comparison of experimental X-ray absorption spectra at the manganese $L_{2,3}$ edge of $[Mn_2O_3]^+$ (blue line) with reference X-ray absorption spectra of $[Mn^{II}{}_2]^+$ cluster (red line) [3] and $[Mn^VO_2]^+$ cluster (yellow line) [5]. The reference spectrum of manganese(V) is shifted by $+0.44 \pm 0.04$ eV to maximize the cosine similarity of the fit of the reference spectra to the X-ray absorption spectrum of $[Mn_2O_3]^+$. The applied shift is physically reasonable and can be expected because of the different coordination number of the manganese(V) centers in $[Mn_2O_3]^+$ and in $[MnO_2]^+$ [6, 8]. Remarkably, the fit of the reference spectra to the spectrum of $[Mn_2O_3]^+$, as indicated by a cosine similarity of 0.994, is almost a perfect match.

In order to evaluate the effectiveness of each pair of reference spectra in replicating the x-ray absorption spectrum of $[Mn_2O_3]^+$, we performed a least-square fit using the `lsqcurvfit` function in Matlab version R2023a. This allowed us

to determine the individual contributions, denoted as a and b, of the reference spectra for both combinations of manganese(II) and manganese(V) as well as manganese(III) and manganese(IV). The resulting ratio for the manganese(II) and manganese(V) pair was found to be 1:0.9. The fits are presented in figure 1 of the main text. To quantify the similarity of the fits and the experimental X-ray absorption spectrum of $[Mn_2O_3]^+$ we rely on cosine similarity, as reported in ref. [9]. We find cosine similarities of 0.983 for the combination of $[Mn^{II}{}_2]^+$ and $[Mn^VO_2]^+$ as reported in the main text, already demonstrating that the metal centers in $[Mn_2O_3]^+$ exist in oxidation states +2 and +5, respectively.

As described in the main text, there is an inherent uncertainty of approximately

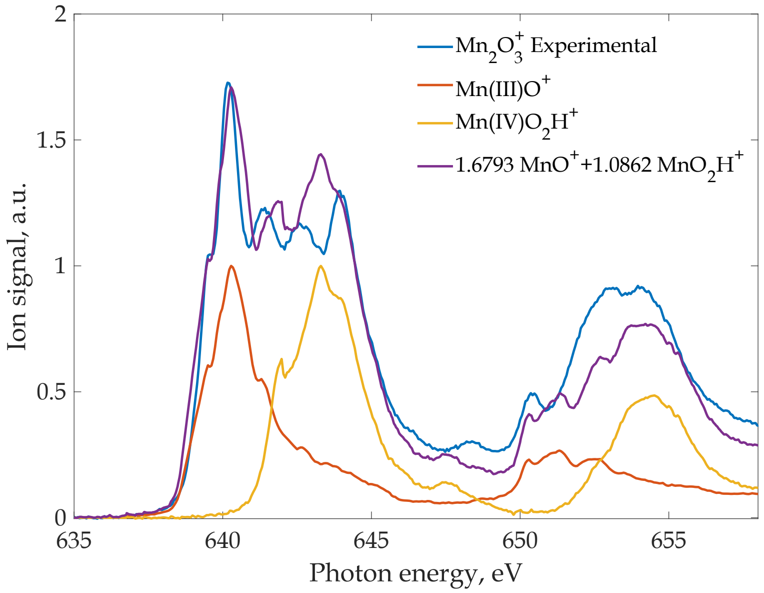

SI Figure 7: Comparison of the experimental manganese $L_{2,3}$ edge spectrum of $[Mn_2O_3]^+$ (blue line) with reference $L_{2,3}$ edge spectra of $[Mn^{III}O]^+$ (red line) [4] and $[Mn^{IV}O_2H]^+$ (yellow line) [5]. The relative shift between the reference spectra of manganese(III) and manganese(III) is 2.36 ± 0.9 eV, resulting from absolute shifts of -0.6 ± 0.08 eV for manganese(III) and of 1.76 ± 0.04 eV for manganese(IV), respectively, to maximize the cosine similarity. The value of cosine similarity is 0.987. Note that the energy shift between the two reference spectrum is 2.6 times the energy shift expected for manganese differing in oxidation state by 1, see SI figure 5.

0.44 eV in the exact energy position of the $L_{2,3}$ excitation energy due to varying

coordination and fractional 3d orbital occupation [8, 6]. To account for this uncertainty, we performed a fitting procedure by shifting the reference spectra by up to 0.44 eV in steps of 0.04 eV and determining their cosine similarity with the experimental spectrum. The best cosine similarity of 0.9941 was achieved for the pair of manganese(II) and manganese(V) reference spectra when a shift of +0.44 eV was applied to the manganese(V) reference spectrum. A shift of the manganese(II) reference spectrum did not improve the cosine similarity further. These spectra are displayed in SI figure 6 showing almost perfect agreement of the spectral shapes of the sum of manganese(II) and manganese(V) reference spectra with that of $[Mn_2O_3]^+$.

Even when there were no physical constraints on the applied shifts, the cosine similarity for the pair of manganese(III) and manganese(IV) reference spectra is never better than 0.987, lower than the value of 0.994 obtained for manganese(II) and manganese(V), with the fit shown in SI figure 7. Note that this results from a large relative shift of the two reference spectra of 2.36 ± 0.9 eV. This resulting shift is more than twice as large as the 0.90 ± 0.09 eV, see SI figure 5, expected for two metal centers differing by one oxidation state only, even when subtracting 0.44 eV to account for the lower coordination number of the manganese(IV) reference compound. This large energy shift indicates that oxidation states of the manganese centers in $[Mn_2O_3]^+$ differ by three, contradicting the initial assumption that oxidation states would differ by only one, for which such a shift is nonphysical.

S3.3 Detailed Discussion of the XMCD Signal and Spin State of $[Mn_2O_3]^+$

Figure 3 of the main text shows the XMCD spectrum of $[Mn_2O_3]^+$, which is the difference in x-ray absorption with helicity of the x-ray beam parallel and antiparallel to the magnetic field axis. The splitting of the leading line at 640 eV in the XMCD spectrum of $[Mn_2O_3]^+$ is presumably due to the reduced local symmetry at the low coordination site of $[Mn_2O_3]^+$ when compared to the metal atoms in $[Mn_2]^+$. This in turn partially lifts the degeneracy of the 3d-derived states, and splits the leading line of the XMCD spectrum as a result. Nonetheless the intense signal in the XMCD of $[Mn_2O_3]^+$ at 640 eV can be attributed to its manganese(II) center.

Moreover, there is also significant negative XMCD intensity at around 644 eV and 654.5 eV that does not correspond to the intensity of the manganese(II) reference, but follows the spectral signature of the high spin manganese(V) reference spec-

trum when inverted in sign, as shown in figure 3 of the main text. Interestingly, the high energy ($\geq$ 642 eV) features of the manganese(II) reference spectrum of Mn_2^+ seem to be absent in $[Mn_2O_3]^+$. This likely originates from the increased covalent bond of the manganese(II) center to the bridging oxygen atoms in $[Mn_2O_3]^+$ in contrast to $[Mn_2^{II}]^+$. The resulting suppression of these features as well as the bunching of the L_2 line and its shift to lower excitation energy can be inferred from Hartree-Fock simulations presented in the following SI section S3.4. From these simulations, the positive intensity of the $[Mn_2O_3]^+$ XMCD signal at 651 eV can again be attributed to the manganese(II) site. Hence, both manganese centers in $[Mn_2O_3]^+$ in oxidation states +2 and +5 are local high spin states, which are coupled antiferromagnetically.

However, the NEVPT2 calculation also predict a sextet state with a local low spin state at the manganese(V) center that is only 0.06 eV above the ground state, below the accuracy of the theory, and can therefore be considered degenerate. The additional presence of $[Mn_2O_3]^+$ in a sextet state can not conclusively be ruled out from the experimental data. If a local low spin state at the manganese(V) center of $[Mn_2O_3]^+$ were to exist, it would show a vanishing contribution to the XMCD signal of the $[Mn_2O_3]^+$ complex. Hence, the XMCD signature of $[Mn_2O_3]^+$ would solely follow the spectral signature of the $[Mn_2^{II}]^+$ XMCD reference spectrum and could not be disentangled from the ground state spectrum. However, for a significant contribution of the sextet state of $[Mn_2O_3]^+$ to the XMCD spectrum, the XMCD spectrum should show only positive intensity above 642 eV, contrary to the experimental findings. Hence, the contribution, if present at all, can be considered minor. Additionally, assuming a vanishing barrier between the quartet and sextet state, the population of the latter would be negligible at typical experimental temperatures of 20 K, based on a relative energy of 0.06 eV.

S3.4 Influence of Hybridization on the XMCD Signature of Manganese(II)

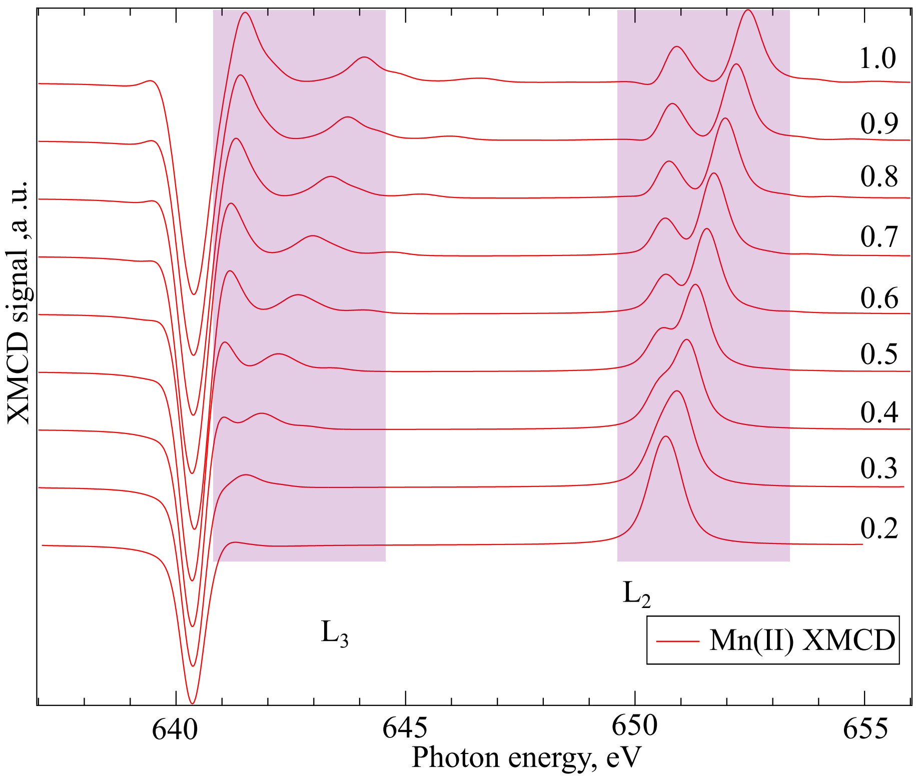

SI Figure 8: Hartree-Fock simulations of atomic manganese(II) with Slater integrals scaled down from 1 to 0.2 to simulate increasing hybridization [10] of the manganese(II) site in $[Mn_2O_3]^+$ with the bridging oxygen atoms. Two main effects can be observed in the highlighted areas: First, the features between 641-644 eV are suppressed with increasing hybridization. Second, the L_2 edge is bunched together resulting in a single band that is also shifted down in energy with increasing hybridization.

We performed Hartree-Fock simulations of the XMCD signal of manganese(II) using the program package CTM4XAS [11]. A Gaussian as well as Lorentzian broadening of 0.2 eV was applied. The aim is to simulate the influence of varying hybridization on the spectral shape of the XMCD signal of manganese(II). As can be seen in SI figure 8, the features between excitation energies 641-644 eV are suppressed with decreasing Slater integral scaling that in turn simulates

increasing hybridization [10]. Furthermore, the L_2 edge becomes more featureless and exhibits only a single band at strong hybridization which is also shifted down in energy.

S4 Computational Procedure to Identify Geometrical and Electronic Ground State of $[Mn_2O_3]^+$

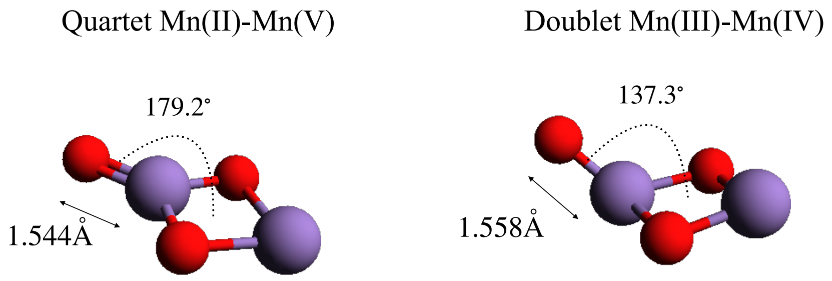

SI Figure 9: Calculated structures of the planar quartet ground and doublet excited states of the $[Mn_2O_3]^+$ complex. Oxygen atoms are shown in red, while manganese atoms are depicted in purple. Additionally, the manganese-to-terminal-oxygen bond length and the angle of the terminal oxygen to the molecular frame is shown. While the manganese centers in the quartet ground state are in oxidation states +2 and +5 they are in oxidation states +3 and +4 in the doublet excited state. Note that at a DFT level of theory these states are swapped and the doublet becomes the ground state, see table 1 of the main text.

A prerequisite for studying the spin state energetics of $[Mn_2O_3]^+$ is to establish its lowest-energy molecular geometry. The multireference character present in $[Mn_2O_3]^+$ necessitates the use of multiconfigurational wavefunction-based methods. However, to date, there are no multiconfigurational computational methods available that would allow for geometry optimizations with sufficiently large active space sizes while also including the dynamical electron correlation. Thus, as a

compromise, we resorted to the use of unrestricted Kohn-Sham density functional theory (UKS-DFT) and broken-symmetry (BS-DFT) methodologies. Based on the success in previous studies [12, 13, 14], the TPSSh functional [15] was utilized in the geometry optimizations.
It is instructive to briefly outline the set of electronic configurations and concomitant spin states that are relevant for $[Mn_2O_3]^+$. When the two manganese centers feature oxidation states of +2 and +5 and the local spins are coupled ferromagnetically, a total spin of S=7/2 or S=5/2 can result depending on whether we have a local high spin or low spin state at the manganese center in oxidation state +5. A total spin of S=3/2 is found if the local high spin of the manganese center in oxidation state +5 is antiferromagnetically coupled to the spin of the manganese(II) center. In the case of manganese(III) and manganese(IV), ferromagnetic and antiferromagnetic coupling coupling of the local spins yields total spins of S=7/2 and S=1/2, respectively. Accordingly, two different spin octet states are possible. Involvement of an oxyl in $[Mn_2O_3]^+$ leads to even more possible states. Most probable among these is a combination of a manganese(IV), a manganese(II) and an oxyl radical. Ferromagnetic coupling of all local spins in this case results in a total spin of S=9/2 while antiferromagnetic coupling of the oxyl spin gives a total spin of S=7/2. Simplified representations of the different electronic states in terms of arrows are given in the third column of Table 1 of the main text.
For our computational study of $[Mn_2O_3]^+$, the cluster geometry was optimized for each aforementioned state separately by means of broken-symmetry DFT. During those optimizations special care was taken to converge to the right state which has been verified in every case by inspection of Mulliken spin populations. Refined relative state energies have been obtained from a combination of the density matrix renormalization group (DMRG) for static and strong correlation and second order n-electron valence state perturbation theory (NEVPT2) that describes dynamic electron correlation effects (see Computational Details of the main text). Spin densities and populations were obtained from configuration-based heatbath-CI (HCI) calculations. [16] The active space of 25 electrons in 20 orbitals employed in all reported DMRG and HCI calculations has been chosen with the ASS1ST scheme. [17, 18] As expected, it encompasses various combinations of Mn d-orbitals and oxygen p-orbitals.
While in the reported DFT ground state [19, 1] the terminal oxygen is tilted out of the molecular plane, the excited state geometric structure is planar. Our DFT calculations confirm the earlier assignment, see SI figure 9, but the relative state energies revert when refined using DMRG-NEVPT2.

S4.1 Coordinates of $[Mn_2O_3]^+$ Isomers and Selected Structural Parameters of $[Mn_2O_3]^+$ and $S_4^{\mathbf{B}}$

Calculated coordinates of all the isomers listed in table 1 of the main text are listed in SI tables 2-8.

SI Table 2: 2S+1=4, manganese(II)-manganese(V), DMRG energy 0 eV, TPSSh energy 0.10 eV

element	x, Å	y, Å	z, Å
Mn	1.6399100000	0.0229450000	-0.2675470000
Mn	-0.8907360000	-0.0998760000	0.1895750000
O	0.1352070000	1.2323920000	0.0194050000
O	-2.4051190000	-0.1680970000	0.4847580000
O	0.2497380000	-1.3275650000	-0.0358910000

SI Table 3: 2S+1=6, manganese(II)-manganese(V), DMRG energy 0.06 eV, TPSSh energy 0.37 eV

element	x, Å	y, Å	z, Å
Mn	1.6420960000	0.0118580000	-0.0482700000
Mn	-0.9448690000	-0.0948220000	-0.0536050000
O	0.0972640000	1.2276320000	-0.0707680000
O	-2.2808560000	-0.1694400000	0.6896050000
O	0.2153640000	-1.3154280000	-0.1266620000

SI Table 4: 2S+1=8, manganese(II)-manganese(V), DMRG energy 0.22 eV, TPSSh energy 0.24 eV

element	x, Å	y, Å	z, Å
Mn	1.6600000000	0.0238100000	-0.2710500000
Mn	-0.8958940000	-0.1001180000	0.1887410000
O	0.1313540000	1.2376910000	0.0206460000
O	-2.4128870000	-0.1684760000	0.4868690000
O	0.2464270000	-1.3331070000	-0.0349060000

SI Table 5: 2S+1=2, manganese(III)-manganese(IV), DMRG energy 0.26 eV, TPSSh energy 0 eV

element	x, Å	y, Å	z, Å
Mn	1.5122410000	0.0095270000	0.0579100000
Mn	-1.0673320000	-0.0980900000	-0.1780820000
O	0.2405440000	1.1738230000	-0.1045280000
O	-2.3015310000	-0.1728160000	0.7701450000
O	0.3450780000	-1.2526450000	-0.1551450000

SI Table 6: 2S+1=8, manganese(III)-manganese(IV), DMRG energy 0.39 eV, TPSSh energy 0.13 eV

element	x, Å	y, Å	z, Å
Mn	1.5206850000	0.0092490000	0.0591540000
Mn	-1.0882850000	-0.0961970000	-0.2229480000
O	0.2343760000	1.1718350000	-0.0907480000
O	-2.2737430000	-0.1744530000	0.7841110000
O	0.3359670000	-1.2506340000	-0.1392690000

SI Table 7: radical, 2S+1=8, manganese(II)-manganese(IV), DMRG energy not available, TPSSh energy 0.82 eV

element	x, Å	y, Å	z, Å
Mn	1.4639520000	-0.0096970000	0.3841580000
Mn	-1.0106250000	-0.1698280000	-0.4789860000
O	-0.0373540000	1.1945830000	-0.1565240000
O	-1.9188670000	-0.0336170000	0.9709170000
O	0.2318940000	-1.3216410000	-0.3292650000

SI Table 8: radical, 2S+1=10, manganese(II)-manganese(IV), DMRG energy not available, TPSSh energy 1.40 eV

element	x, Å	y, Å	z, Å
Mn	1.4639520000	-0.0096970000	0.3841580000
Mn	-1.0106250000	-0.1698280000	-0.4789860000
O	-0.0373540000	1.1945830000	-0.1565240000
O	-1.9188670000	-0.0336170000	0.9709170000
O	0.2318940000	-1.3216410000	-0.3292650000

S4.2 Comparison of the Electronic Structure of the Manganese(V) center in $[Mn_2O_3]^+$ and the Dangling Manganese in S_4^B

SI Table 9: Comparison of in plane manganese-oxygen bond distances d and manganese-oxygen-oxygen bond angles of the dangling manganese in S_4^B [20] and the manganese(V) site in $[Mn_2O_3]^+$ (this work).

parameter	S_4^B	$[Mn_2O_3]^+$
$\angle$(Mn, O1, O2)	124.88°	98.6°
$\angle$(Mn, O2, O3)	118.03°	131°
$\angle$(Mn, O3, O1)	117.03°	130.4°
d(Mn, O1)	1.726 Å	1.69 Å
d(Mn, O2)	1.749 Å	1.69 Å
d(Mn, O3)	1.61 Å	1.544 Å

While the lowest-energy structure of $[Mn_2O_3]^+$ bears a close structural similarity to the dangling manganese site of OEC in S_4^B, it is lower coordinated and lacks any ligands or substrate water molecules that are present in OEC. In $[Mn_2O_3]^+$ and S_4^B, the local symmetry at the manganese(V) site is reduced by distortions from D_{3h} to C_{2v}, with planar trigonal structure in $[Mn_2O_3]^+$ but trigonal bipyramidal structure in S_4^B [21, 20], see SI table 9 for bond lengths and angles. Different symmetries of the occupied orbitals of the manganese(V) centers in $[Mn_2O_3]^+$ and S_4^B lead to differences in the local electronic structure: Because of the absence of ligands normal to the plane defined by the molecular frame of $[Mn_2O_3]^+$, the molecular orbital of a_1 character ($3d_{z^2}$) is pushed down in energy and becomes almost degenerate with the molecular orbital of a_2 ($3d_{yz}$) symmetry. Hence, manganese in oxidation state +5 in $[Mn_2O_3]^+$ is characterized by a local ($a_1^1 a_2^1$) $^3A_2$ state, while manganese(V) in S_4^B - derived from electron removal in S_3^B, [21] - is in a local ($a_2^1 b_1^1$) $^3B_2$ state. The orbital order of the model complex could, however, be varied by tailoring the local symmetry of the manganese(V) center with appropriate ligands, in order to more closely mimick the orbital order of the dangling manganese site in the S_4^B state.

S5 Localized DMRGSCF Orbitals

At the DMRGSCF level, the spin densities and hence, population analysis were not available to us due to technical limitations. Therefore, we rely on localized active orbitals to deduce the oxidation states of the different spin states of $[Mn_2O_3]^+$. Seven of the nearly singly occupied manganese d-orbitals were localized using the Pipek-Mezey method [22] in ORCA5.0.3.[23, 24] The resulted localized orbitals - that can be considered occupied orbitals - are displayed in SI figures 10-13.

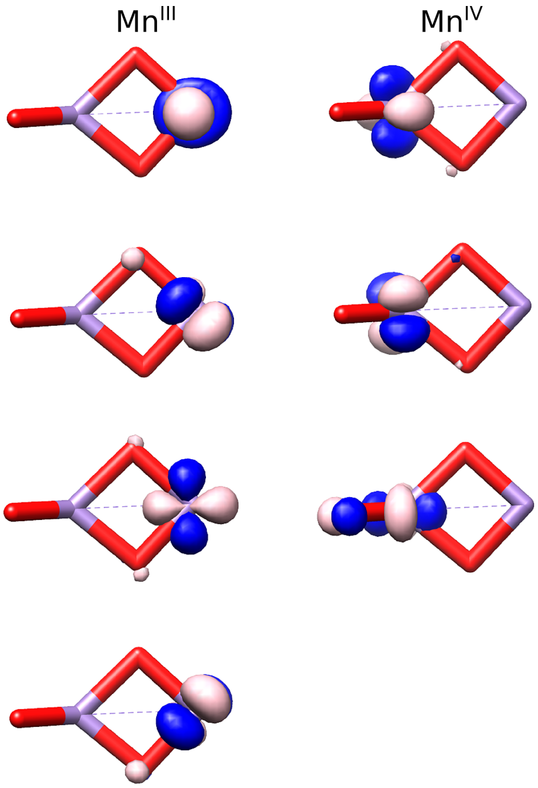

SI Figure 10: Localized DMRGSCF active orbitals of the doublet state (DFT ground state, DMRG-NEVPT2 excited state) of $[Mn_2O_3]^+$. Four 3d orbitals are localized at the low coordinated manganese center, while three 3d orbitals are at the highly coordinated manganese site. Hence, the oxidation states +3 and +4 are assigned.

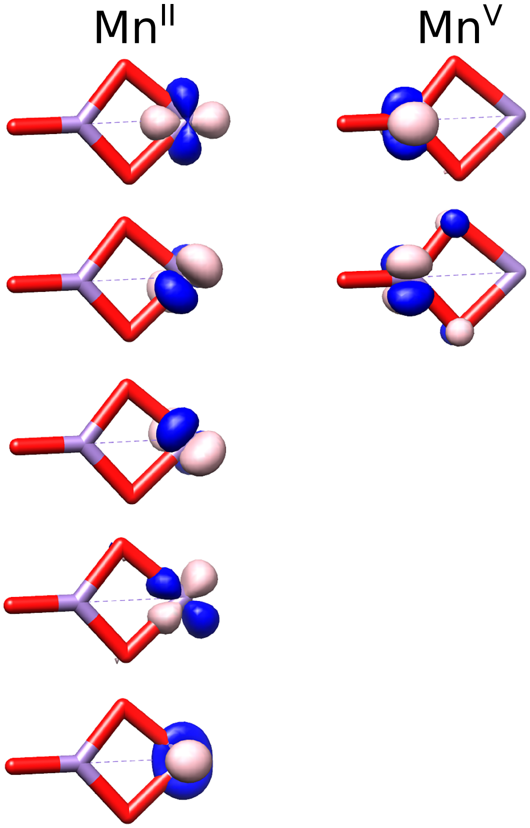

SI Figure 11: Localized DMRGSCF active orbitals of the quartet state (DFT excited state, DMRG-NEVPT2 ground state) of $[Mn_2O_3]^+$. Five 3d orbitals are localized at the low coordinated manganese center, while two 3d orbitals are at the highly coordinated manganese site. Hence, the oxidation states +2 and +5 are assigned.

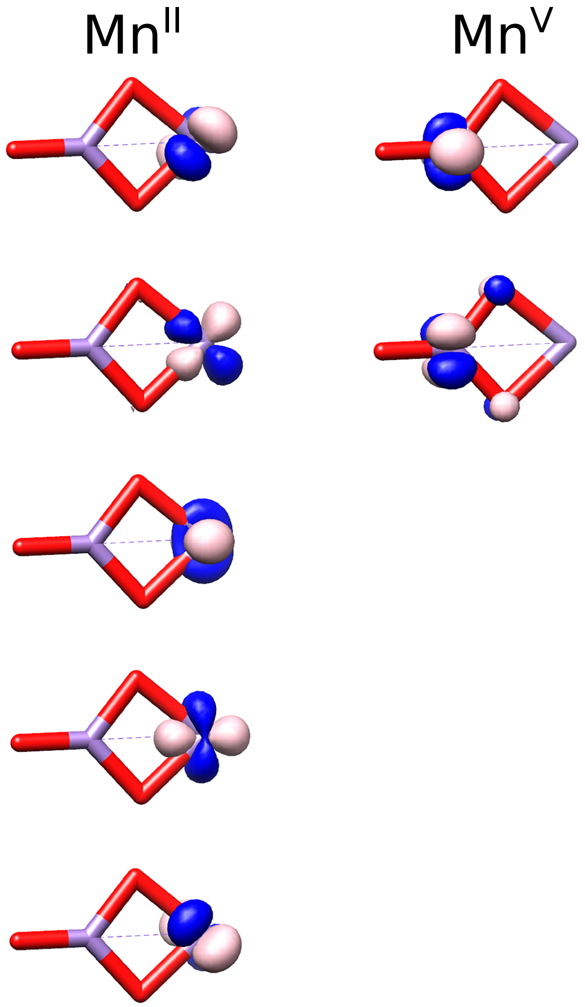

SI Figure 12: Localized DMRGSCF active orbitals of the sextet state of $[Mn_2O_3]^+$. Five 3d orbitals are localized at the low coordinated manganese center, while two 3d orbitals are at the highly coordinated manganese site. Hence, the oxidation states +2 and +5 are assigned.

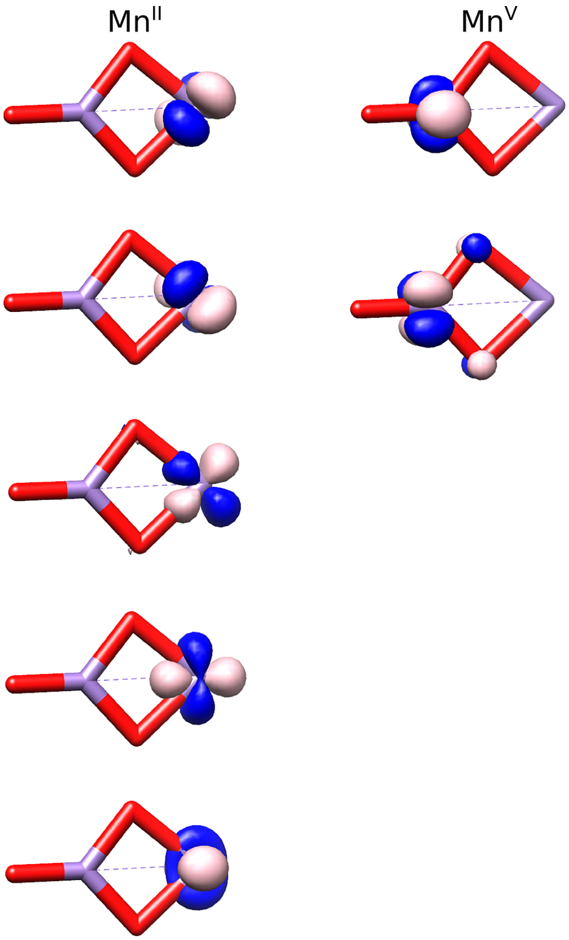

SI Figure 13: Localized DMRGSCF active orbitals of the octet state of $[Mn_2O_3]^+$. Five 3d orbitals are localized at the low coordinated manganese center, while two 3d orbitals are at the highly coordinated manganese site. Hence, the oxidation states +2 and +5 are assigned.

References

[1] Nina Zimmermann, Thorsten M. Bernhardt, Joost M. Bakker, Uzi Landman, and Sandra M. Lang. Infrared photodissociation spectroscopy of dimanganese oxide cluster cations. *Phys. Chem. Chem. Phys.*, 21:23922–23930, 2019.

[2] Silvia Mauthe, Irene Fleischer, Thorsten M. Bernhardt, Sandra M. Lang, Robert N. Barnett, and Uzi Landman. A Gas-Phase CanMn(4-n)O4^+ Cluster Model for the Oxygen-Evolving Complex of Photosystem II. *Angew. Chem. Int. Ed.*, 58(25):8504–8509, 2019.

[3] V. Zamudio-Bayer, K. Hirsch, A. Langenberg, M. Kossick, A. Ławicki, A. Terasaki, B. v. Issendorff, and J. T. Lau. Direct observation of high-spin states in manganese dimer and trimer cations by x-ray magnetic circular dichroism spectroscopy in an ion trap. *J. Chem. Phys.*, 142(23):234301, 2015.

[4] Mickaël G. Delcey, Rebecka Lindblad, Martin Timm, Christine Bülow, Vicente Zamudio-Bayer, Bernd von Issendorff, J. Tobias Lau, and Marcus Lundberg. Soft x-ray signatures of cationic manganese–oxo systems, including a high-spin manganese(v) complex. *Phys. Chem. Chem. Phys.*, 24:3598–3610, 2022.

[5] Olesya S. Ablyasova, Vicente Zamudio-Bayer, Max Flach, Mayara da Silva Santos, J. Tobias Lau, and Konstantin Hirsch. Direct spectroscopic evidence for the high-spin state of dioxidomanganese(v). *Phys. Chem. Chem. Phys.*, 26:5830–5835, 2024.

[6] Max Flach, Konstantin Hirsch, Martin Timm, Olesya S. Ablyasova, Mayara da Silva Santos, Markus Kubin, Christine Bülow, Tim Gitzinger, Bernd von Issendorff, J. Tobias Lau, and Vicente Zamudio-Bayer. Iron L3-edge energy shifts for the full range of possible 3d occupations within the same oxidation state of iron halides. *Phys. Chem. Chem. Phys.*, 24(33):19890–19894, 2022.

[7] Haiyan Tan, Jo Verbeeck, Artem Abakumov, and Gustaaf Van Tendeloo. Oxidation state and chemical shift investigation in transition metal oxides by EELS. *Ultramicroscopy*, 116:24–33, 2012.

[8] Olesya S. Ablyasova, Meiyuan Guo, Vicente Zamudio-Bayer, Markus Kubin, Tim Gitzinger, Mayara da Silva Santos, Max Flach, Martin Timm, Marcus Lundberg, J. Tobias Lau, and Konstantin Hirsch. Electronic Structure

of the Complete Series of Gas-Phase Manganese Acetylacetonates by X-ray Absorption Spectroscopy. *J. Phys. Chem. A*, 127(34):7121–7131, 2023.

[9] Meiyuan Guo, Erik Källman, Rahul V. Pinjari, Rafael C. Couto, Lasse Kragh Sørensen, Roland Lindh, Kristine Pierloot, and Marcus Lundberg. Fingerprinting electronic structure of heme iron by ab initio modeling of metal l-edge x-ray absorption spectra. *J. Chem. Theory Comput.*, 15(1):477–489, 2019.

[10] G. Peng, J. van Elp, H. Jang, L. Jr. Que, W. H. Armstrong, and S. P. Cramer. L-edge x-ray absorption and x-ray magnetic circular dichroism of oxygen-bridged dinuclear iron complexes. *J. Am. Chem. Soc.*, 117(9):2515–2519, 1995.

[11] Eli Stavitski and Frank M.F. F. de Groot. The CTM4XAS program for EELS and XAS spectral shape analysis of transition metal L edges. *Micron*, 41(7):687–694, 2010.

[12] Kasper P Jensen. Bioinorganic chemistry modeled with the tpssh density functional. *Inorg. Chem.*, 47(22):10357–10365, 2008.

[13] Mark A Iron and Trevor Janes. Evaluating transition metal barrier heights with the latest density functional theory exchange–correlation functionals: The mobh35 benchmark database. *J. Phys. Chem. A*, 123(17):3761–3781, 2019.

[14] Chaoyue Zhao, Rongkai Wu, Shuoqing Zhang, and Xin Hong. Benchmark study of density functional theory methods in geometry optimization of transition metal–dinitrogen complexes. *J. Phys. Chem. A*, 127(32):6791–6803, 2023.

[15] John P Perdew, Jianmin Tao, Viktor N Staroverov, and Gustavo E Scuseria. Meta-generalized gradient approximation: Explanation of a realistic nonempirical density functional. *J. Chem. Phys.*, 120(15):6898–6911, 2004.

[16] Mihkel Ugandi and Michael Roemelt. A configuration-based heatbath-ci for spin-adapted multireference electronic structure calculations with large active spaces. *J. Comput. Chem.*, 44:2374–2390, 2023.

[17] Abhishek Khedkar and Michael Roemelt. Active space selection based on natural orbital occupation numbers from n-electron valence perturbation theory. *J. Chem. Theory Comput.*, 15(6):3522–3536, 2019.

[18] Abhishek Khedkar and Michael Roemelt. Extending the ass1st active space selection scheme to large molecules and excited states. *J. Chem. Theory Comput.*, 16(8):4993–5005, 2020.

[19] G. L. Gutsev, K. V. Bozhenko, L. G. Gutsev, A. N. Utenyshev, and S. M. Aldoshin. Dependence of Properties and Exchange Coupling Constants on the Charge in the Mn2O n and Fe2O n Series. *J. Phys. Chem. A*, 122(25):5644–5655, 2018.

[20] Vera Krewald, Frank Neese, and Dimitrios A. Pantazis. Implications of structural heterogeneity for the electronic structure of the final oxygen-evolving intermediate in photosystem II. *J. Inorg. Biochem.*, 199:110797, 2019.

[21] Marius Retegan, Vera Krewald, Fikret Mamedov, Frank Neese, Wolfgang Lubitz, Nicholas Cox, and Dimitrios A. Pantazis. A five-coordinate Mn(IV) intermediate in biological water oxidation: Spectroscopic signature and a pivot mechanism for water binding. *Chem. Sci.*, 7(1):72–84, 2016.

[22] János Pipek and Paul G Mezey. A fast intrinsic localization procedure applicable for ab initio and semiempirical linear combination of atomic orbital wave functions. *J. Chem. Phys.*, 90(9):4916–4926, 1989.

[23] Frank Neese. The orca program system. *Wiley Interdiscip. Rev.: Comput. Mol. Sci.*, 2(1):73–78, 2012.

[24] Frank Neese, Frank Wennmohs, Ute Becker, and Christoph Riplinger. The orca quantum chemistry program package. *J. Chem. Phys.*, 152(22), 2020.
